# Thickness of glomerular and tubular basement membranes in preclinical and clinical stages of diabetic nephropathy

**DOI:** 10.4103/0971-4065.42336

**Published:** 2008-04

**Authors:** I. Tyagi, U. Agrawal, V. Amitabh, A. K. Jain, S. Saxena

**Affiliations:** Institute of Pathology, ICMR, Post Box 4909, India; 1 Safdarjung Hospital, New Delhi- 110 029, India

**Keywords:** Glomerular basement membrane, microalbuminuria, morphometry

## Abstract

**Aims:**

This study aimed to elucidate the early renal changes in diabetes mellitus (DM) with and without clinical symptoms related to renal damage.

**Methods:**

Renal biopsy was studied in 25 patients (14 with microalbuminuria and 11 with albuminuria) both by light and electron microscopies (LM and EM, respectively) for renal changes and morphometry was performed to study glomerular and tubular basement membranes (GBM and TBM, respectively) width using a Soft Imaging System GmBH (analysis 3).

**Results:**

A significant increase was noted in the mean GBM and TBM thickness in both the preclinical and clinical groups compared to the control group. The changes in the TBM were noted to be predominant in both preclinical and clinical patients.

**Conclusions:**

This study indicates the importance of morphometric evaluation of the GBM and TBM width in the elucidation of early renal damage in diabetic nephropathy, especially in the absence of LM changes. The significance of identification of early renal changes using morphometric techniques for better management of these patients requires further studies.

## Introduction

Diabetes Mellitus (DM) is a metabolic disease characterized by absolute or relative insulin deficiency due either to inadequate secretion or insulin resistance. Type 1 DM is usually associated with absolute insulin deficiency caused by autoimmune destruction of insulin-producing cells in the pancreas, while type 2 diabetes or non-insulin-dependent diabetes mellitus (NIDDM) is characterized by insulin resistance in peripheral tissue and an insulin secretory defect in the beta cells.

Diabetic nephropathy is a frequent complication of DM and occurs with either type 1 or type 2 diabetes, particularly in patients with poor glycemic control. However, the natural history and pathology are similar in both types. The various risk factors associated with development of diabetic nephropathy are the duration of the disease, gender, racial and ethnic factors, genetic factors and degree of metabolic control.^1^ Although India is termed the ‘Diabetes capital of the world’ the prevalence of microvascular complications such as retinopathy and nephropathy are comparatively lower. The prevalence of overt nephropathy has been reported to be 2.2% and that of microalbuminuria to be 26.9%.^2^

It is a well-accepted fact that metabolic control plays an important role in the development of diabetic nephropathy (DN). The early phase of nephropathy is characterized by microalbuminuria (MA, 30-300 µg/24 h), which can be recorded by sensitive laboratory measurements and this is followed by overt albuminuria (>300 µg/24 h). There is increased risk of microalbuminuria with a longer duration of diabetes and higher glycosylated hemoglobin value.^3^

Since the spectrum of morphological changes in DM patients with clinical proteinuria have been well characterized, renal changes associated with microalbuminuria have not been well delineated. Since light microscopic (LM) examination of renal biopsy does not reveal significant changes at this stage, we conducted ultrastructural study of glomerular changes and morphometric analysis of glomerular and tubular basement membranes (GBM and TBM, respectively) to characterize progressive renal changes.

## Materials and Methods

In this study, 47 patients with type 2 DM referred from the Department of Nephrology, Safdarjung Hospital, New Delhi, from October 2003 to April 2005 were included. A detailed history was acquired for every patient. The onset of diabetes was defined as the time when the patient first visited with symptoms such as thirst and polyuria and when either urinary sugar or hyperglycemia was first detected. Approval for the study was granted by the ethics committee of Safdarjung Hospital and consent was obtained from each patient.

Diabetes was diagnosed according to the World Health Organization (WHO) criteria.^4^ Fasting and postprandial plasma glucose were measured by oxidase method. Urinary albumin and sugar were estimated by dipstick method. Of the 47 patients, 11 had albuminuria (A) and were included in the clinical group. The remaining 36 patients were subjected to microalbuminuria estimation by radioimmunoassay. Microalbuminuria (MA) was detected in a total of 14 patients who were included in the preclinical category. The 25 patients with albuminuria and/or microalbuminuria were investigated further for HbA1c, blood urea, serum creatinine and renal biopsy.

Three renal tissue cores were collected in 4% formaldehyde, 70% ethanol and forglu for LM, immunofluorescence and electron microscopy (EM), respectively. To facilitate histological diagnosis, special stains such as Periodic acid-Schiff (PAS), silver methenamine and Masson's trichrome were used besides hematoxylin and eosin (H & E). Direct immunofluorescence staining of frozen sections was performed for immunoglobulin (Ig)A, IgG, IgM, complement 3 (C3) and fibrinogen. For EM studies, ultra-thin sections were stained with aqueous saturated solution of uranyl acetate and lead citrate. These contrasted sections on the grid were examined under HITACHI H7500 TEM. The sections were studied for ultrastructural changes in the glomeruli, tubules and blood vessels.

**Fig. 1:**
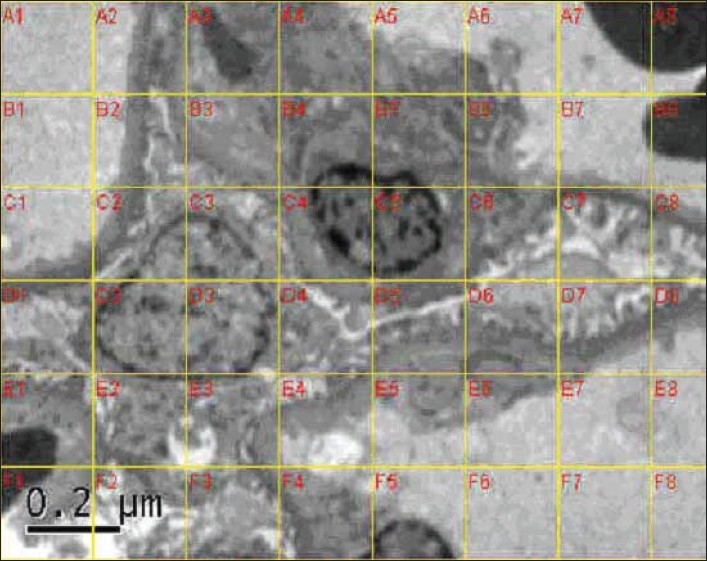
Ultrastructure of the glomerular capillary loop with a grid overlay for morphometric assessment

A series of images covering the entire available glomeruli and tubules were obtained through Gatan charge-coupled device (CCD) camera at a magnification of 6000x; these images were then converted to JPEG format to perform morphometric analysis to measure the GBM and TBM thickness by using a Soft Imaging System GmbH (analysis 3). The images were calibrated using an inbuilt micron bar. A 200 × 200 nm grid was overlaid on the images[Fig.1]. GBM and TBM width was obtained by the orthogonal intercept method. The measurements were made at each point where a grid line intercepted an endothelial/peripheral GBM interface. The GBM width was measured on a line orthogonal to the edge of the peripheral GBM at the endothelial side of the intercept. TBM was measured at 50 orthogonal intercepts per patient and presented as mean ± SD.

Seven patients of age-matched minimal change nephrotic syndrome (MCNS) were used as the control for comparison. The mean GBM and TBM thickness was then calculated for each patient in all the three patient groups [control, preclinical (MA) and clinical (A) groups]. Then, the mean GBM and TBM thickness was calculated in each of the three groups and the data was presented as mean values ± SD.

The data were analyzed for statistical differences among different groups by using Statgraphics Statistical Package. Student's t test was applied to estimate the statistical variation between the means and variances of two samples; chi-Square test was applied to determine the effect of different conditions on different parameters.

## Results

The biochemical parameters in the preclinical and clinical groups are presented in Table 1 and show a significantly longer duration of disease, higher blood urea and serum creatinine levels in the clinical group than that in the preclinical group.

**Table 1:** Biochemical parameters in cohort

Biochemical parameter	Preclinical n (%) n = 14	Clinical n (%) n = 11	*P* value
**Duration** (years)			
0–5	9(64.3)	0	0.002
6–10	5(35.7)	8(72.7)	
>10	0	3(27.3)
**Fasting blood glucose** (mg%)			
80–110	4(28.6)	1(9.1)	
111–200	9(64.3)	6(54.5)	0.14
>200	1(7.1)	4(36.4)	
**Postprandial blood glucose** (mg%)			
100–140	2(14.3)	0	
141–240	11(78.6)	7(63.6)	0.11
>240	1(7.1)	4(36.4)	
**HbA1c**(%)			
<7	4(28.6)	2(18.2)	
≥7	10(71.4)	9 (81.8)	0.9
**Blood urea** (mg%)			
<35	14(100)	4(36.4)	
≥35	0	7(63.6)	0.002
**Serum creatinine** (mg%)			
<1.5	14(100)	3(27.3)	
≥1.5	0	8(72.7)	0.001

### Morphological features

LM alterations predominated in the clinical group (90.91%) compared to those in the preclinical group (21.43%) [Table 2]. LM characteristics of diffuse glomerulosclerosis (GS) were noted in only three patients (21.43%) of the preclinical group and the remaining 11 (78.57%) patients showed normal morphology. In the clinical group, five (45.45%) patients showed the characteristics of diffuse glomerulosclerosis, four (36.36%) showed the characteristics of both diffuse and nodular GS, one (9.09%) of nodular GS and one (9.09%) showed no significant morphological alteration. Two of these patients showed a capsular drop adherent to the Bowman's capsule and 2 patients showed a hyaline cap in the capillary loops. Tubular degeneration, atrophy, hyaline casts and interstitial fibrosis were noted in most of these patients. TBM was observed to be thickened in most of the clinical groups.

**Table 2:** Characteristics of the study group

**Total number of patients with diabetes**	47
Normoalbuminuric n (%)	22
Microalbuminuric n (%)	14 (56%)
Diffuse Glomerulosclerosis	3(21.43%)
Diffuse and nodular glomerulosclerosis	0
Nodular glomerulosclerosis	0
Normal morphology	11(78.57)
Albuminuric n (%)	11(44%)
Diffuse glomerulosclerosis	5(45.45%)
Diffuse and nodular glomerulosclerosis	4(36.37%)
Nodular glomerulosclerosis	1(9.09%)
Normal morphology	1(9.09%)
**GBM thickness (mean ± SD in nm)**	
Preclinical (MA)	464.66 ± 95.9
Clinical	578.37 ± 192.29
Control	309.00 ± 40.48
*P* value (between control and either patient groups)	<0.005
**TBM thickness (mean ± SD in nm)**	
Preclinical (MA)	615.62 ± 97.41
Clinical	752.42 ± 156.87
Control	399.7 ± 33.72
*P* value (between control and either patient groups)	<0.005

### Ultrastructural features

Among 11 preclinical patients showing normal morphology on LM, 9 showed thickening in both GBM and TBM, effacement of foot processes, widening of filtration slits and mild mesangial widening. Thickening of the vessel wall was noted in four of these patients. The remaining two patients showed thickness of the GBM and TBM, which was similar to that noted in control patients with no other ultrastructural changes. HbA1c was however less than 7 in both patients and the duration of diabetes was more than five years. All the three preclinical and 10 clinical patients that showed diffuse or nodular GS on LM showed diffuse or segmental thickening of GBM and TBM, the presence of homogenous electron-dense material between the endothelial cells and the GBM (early hyalinosis or exudative lesions) as well as in the TBM in one patient. All patients showed mesangial widening and hypercellularity, variable effacement of foot processes and widening of filtration slits. The presence of electron-dense material between the Bowman's capsule and parietal epithelium was evident in the patients showing a capsular drop. Thickening of vessel walls was also noted in four patients. In contrast, one patient in the clinical group with normal histology showed thinning of GBM and TBM along with other ultrastructural findings.

### Morphometry

The mean ultrastructural thicknesses of GBM and TBM were compared in the preclinical, clinical and control (MCNS) groups; these values showed a significant increase from the control to the preclinical and further to the clinical group, suggesting that the thickness of GBM and TBM play a significant role in proteinuria.

The mean GBM and TBM thickness in MA patients with LM changes (493.81 ± 95.92 nm and 562.02 ± 181.16 nm, respectively) were found to be increased compared to that in the control group (309.00 ± 40.48 nm). Although two of these patients (2/11) showed thickness of both GBM and TBM, which was similar to that in the controls [Table 3], the remaining 9 patients exhibited GBM thickness (*P* < 0.05) comparable to the three patients who had LM changes, indicating that the ultrastructural changes, especially the thickness of both GBM and TBM is an early marker of renal damage. The three MA patients showing LM changes had significantly higher mean GBM and TBM thickness of 562.02 ± 181.16 nm and 687.17 ± 159.52 nm, respectively.

**Table 3:** Morphometric evaluation of subgroups

Parameter	Number of patients	GBM thickness	TBM thickness
**HbA1c**			
Preclinical (MA)			
<7	4	436.0 ± 85.958	580.22 ± 81.833
≥7	10	536.0 ± 90.231	693.50 ± 91.398
*P* value		0.08	0.05
Clinical (A)			
<7	2	529.04 ± 171.697	709.44 ± 137.838
≥7	9	800.10 ± 116.955	942.50 ± 71.418
*P* value		0.07	0.05
**Duration (years)**			
Preclinical (MA)			
0–5	9	413.90 ± 72.642	555.38 ± 61.718
6–10	5	555.86 ± 56.037	710.60 ± 57.808
>10	0	0	0
*P* value (between the I & II groups)		0.003	0.001
Clinical (A)			
0–5	0	0	0
6–10	8	504.13 ± 165.239	685.75 ± 126.249
>10	3	3 776.20 ± 92.482	928.00 ± 56.400
*P* value (between the I & II groups)		0.03	0.01
**Morphology**			
Preclinical (MA)			
No LM changes present (MA − LM)	11	493.81 ± 95.92	562.02 ± 181.16
LM changes present (MA + LM)	3	562.02 ± 181.16	687.17 ± 159.52
*P* value		<0.005	<0.005
Clinical (A)		
No LM changes present (A − LM)	1	362.08 ± 30.17	515.60 ± 16.67
LM changes present (A + LM)	10	600 ± 188.06	776.11 ± 143.14

The clinical group showed a significantly higher mean GBM thickness (578.37 ± 192.29 nm) compared to that in the preclinical (464.66 ± 95.9 nm; *P* < 0.005) and control groups (309.00 ± 40.48 nm; *P* < 0.005). These observations suggest that the GBM thickness increases with the progress from the preclinical to clinical stage. Similarly, it was observed that the TBM thickness was lower in the control group (399.7 ± 33.72 nm) and increased significantly as the patient progressed from the preclinical (615.62 ± 97.41 nm) to clinical stage (752.42 ± 156.87 nm; *P* = 0.005). Only one patient in the clinical group showed normal morphology and thickness of the GBM compared to the control group.

A direct correlation of both GBM and TBM thickness has been noticed with a high HbA1C (>7%); however, this increase was found to be significant in TBM (*P* = 0.05) only. The thickness of the GBM and TBM was also found to be correlated with the duration of the disease; increased thickness is observed in patients with longer duration than those with shorter duration (*P* = 0.005) [Table 3].

The mean thickness of GBM and TBM has been noticed to be considerably lower in the control (MCNS), microalbuminuric and albuminuric patients in our study group than those observed in the Caucasian populations [Table 4].

**Table 4:** Comparative morphometry of GBM and TBM

	Our study	Drummond *et al*^11^	Osterby *et a1*l2	Caramori *et al*^13^	Rayat *et al*^14^	Brito *et al*^15^
**GBM**						
Preclinical	464.66 ± 95.9	510 ± 93	–	602 ±157	–	–
Clinical	578.37 ± 192.3	–	562	700 ± 141	–	–
Control	309.1 ± 33.7	425 ± 78	353	332 ± 46	321 ± 28	–
*P* value	<0.005	0.003	0.0001	<0.0001	–	–
(Control vs patient)						
**TBM**						
Preclinical	615.6 ± 97.4	–	–	–	–	915 ± 320
Clinical	752.42 ± 156.8	–	–	–	–	–
Control	399.7 ± 33.72	–	–	–	–	558 ± 116
*P* value	<0.005	–	–	–	–	0.0005
(Control vs patient)					

## Discussion

Renal disease is the leading cause of death and disability in diabetes. GS is the most important cause of renal failure in diabetic patients since approximately 30% of the diabetic patients die due to renal failure.^5^ A high proportion of individuals with type 2 DM are found to have microalbuminuria and overt nephropathy shortly after diagnosis since diabetes is actually present for many years before being diagnosed.^6^ Without specific interventions, 20–40% of type 2 diabetic patients with microalbuminuria progress to overt nephropathy.^7^ If diabetic patients do not have proteinuria after 25–30 years of diabetes, they are unlikely to ever develop GS. Once proteinuria occurs, progression to end-stage renal disease (ESRD) is inevitable despite good metabolic control.^8^,^9^ It is therefore imperative to recognize the onset of renal damage to plan further management to reverse the progression of this disease. At present, MA is the most reliable feature to predict early renal damage in diabetic patients.

The evolution of diabetic GS is described in five clinical stages. The initial two stages include increased GFR with hypertrophic kidneys and a stage in which glomerular lesions develop in the absence of clinical evidence of renal disease. During these stages, the GBM begins to thicken and the mesangial matrix expands. These two stages are reversible with a good glycemic control. This is followed by stages 3–5 manifested by the development of microalbuminuria, overt diabetic nephropathy and ESRD, respectively.^10^ The incidence of diabetic GS is known to increase with the duration of diabetes, and our study has demonstrated a significant increase in BM thickness with increasing duration, irrespective of whether the patient had microalbuminuria or albuminuria. The GBM and TBM width were also found to increase with HbA1c levels but not with fasting and postprandial glucose levels, indicating the influence of consistent glycemic control in the occurrence of basement membrane thickness.

Drummond *et al.*^11^ had demonstrated that the mean GBM thickness was significantly higher in type I diabetic microalbuminuric patients (510 ± 93 nm) as compared to the controls (425 ± 78 nm), while Osterby *et al.*^12^ showed a similar increase in albuminuric patients (562 nm) compared to the controls (353 nm). In our study group comprising mainly type 2 diabetic patients with microalbuminuria (preclinical), a significantly large number of patients (78.6%) had normal renal morphology as observed by LM, and only one patient with overt nephropathy showed normal morphology. Ultrastructural examination revealed a large number of patients (48%) with increased GBM membrane thickness in both the preclinical and clinical groups even without LM change.

Caramori *et al.*^13^ demonstrated a progressively increasing GBM width in type 1 diabetic patients as protein excretion progressed from normoalbuminuria to microalbuminuria and further to proteinuria; these findings were consistent with those of the present study, indicating the importance of morphometry in evaluating GBM and TBM width for the possible diagnosis of early diabetic nephropathy especially in the event of no microscopic changes. Ultrastructural morphometry showed GBM and TBM thickness in 64.3% patients, while 14.3% did not show any changes in either GBM or TBM, suggesting that with a good glycemic control, the progression to renal damage could be prevented.

In this study, the TBM thickness was higher than that of the GBM. This could possibly be due to the presence of tubular atrophy. TBM thickening has previously been reported in diabetics compared to nondiabetics. Vascular changes were observed under LM but not measured on EM.

A population difference has been noted in the mean thickness of GBM and TBM in all groups of Indian patients compared to the findings in Caucasians; however, this finding needs to be confirmed using a larger set of samples. A systematic study on GBM thickness by Rayat *et al.* in the Indian population showed the mean GBM thickness to be 321 ± 28 nm.^14^ This value is comparable to that reported by us in this study. It is possible that the genes mediating the synthesis of capillary GBM and mesangial matrix components have ethnic variation accounting for variation in basement membrane thickness and also causing a delay in the progression to diabetic glomerulosclerosis. The significance of identification of early renal changes using morphometric techniques is that it may enable reversing of the renal damage by controlling microalbuminuria.^15^
